# Associations between matrix metalloproteinase gene polymorphisms and glaucoma susceptibility: a meta-analysis

**DOI:** 10.1186/s12886-017-0442-2

**Published:** 2017-04-21

**Authors:** Ming-Yue Wu, Yang Wu, Yong Zhang, Cai-Yun Liu, Chun-Yan Deng, Le Peng, Lan Zhou

**Affiliations:** 1 Stomatologic Hospital & College, Anhui Medical University, Key Laboratory of Oral Diseases Research of Anhui Province, Hefei, 230032 China; 2 Department of Neurology, Center for Evidence-Based Medicine and Clinical Research, Taihe Hospital, Hubei University of Medicine, 32 South Renmin Road, Shiyan, 442000 China; 3 Department of Oral and Maxillofacial Surgery, State Key Laboratory Breeding Base of Basic Science of Stomatology & Key Laboratory of Oral Biomedicine Ministry of Education, School and Hospital of Stomatology, Wuhan University, Wuhan, China; 4 Department of Ophthalmology, Taihe Hospital, Hubei University of Medicine, Shiyan, 442000 China; 5 Department of Stomatology, Taihe Hospital, Hubei University of Medicine, 32 South Renmin Road, Shiyan, 442000 China; 6 Intensive Care Unit, Taihe Hospital, Hubei University of Medicine, Shiyan, 442000 China

**Keywords:** Matrix metalloproteinases, Glaucoma, Polymorphism

## Abstract

**Background:**

Matrix metalloproteinases (MMPs) polymorphisms have been implicated in the pathogenesis of glaucoma risk. However, the results were controversial. We performed a meta-analysis to evaluate the precise associations between MMPs polymorphisms and glaucoma risk.

**Methods:**

Related studies were reviewed by searching electronic databases within four databases. Odds ratios (ORs) with 95% confidence intervals (CIs) were calculated to assess the association between the most common polymorphisms of MMPs and glaucoma risk. Heterogeneity, publication bias and sensitivity analysis were conducted to guarantee the statistical power.

**Results:**

Overall, 11 selected articles involving 2,388 cases and 2,319 controls were included in this meta-analysis. Significant associations were only found between MMP-9 rs17576 G > A polymorphism (GA vs. GG: OR = 0.80, 95%CI = 0.67-0.97, *P* = 0.02, I^2^ = 0%), MMP-9 rs3918249 C > T polymorphism (TT vs. CC + CT: OR = 0.71, 95%CI = 0.51-0.98, *P* = 0.04, I^2^ = 0%) and glaucoma risk in the general population. Subgroup analysis also suggested that MMP-9 rs17576 G > A was related to glaucoma in the Caucasian population (GA vs. GG: OR = 0.67, 95%CI = 0.45-1.00, *P* = 0.05; GA + AA vs. GG: OR = 0.66, 95%CI = 0.45-0.97, P = 0.03, I^2^ = 0%).

**Conclusions:**

Our meta-analysis demonstrates that MMP-9 rs17576 G > A polymorphism might be a protective factor against the development of glaucoma in Caucasian population.

## Background

Glaucoma is a heterogeneous disease of the eye characterized by the progressive degeneration of retinal ganglion cells and loss of vision associated with elevated intraocular pressure (IOP) [1]. After cataracts, glaucoma is the second leading cause of blindness in the world [2]. In China, there were approximately 15.8 million patients with glaucoma in 2010 and the number of patients is projected to increase to 21.8 million by 2020 [3]. This visual disorder results in severe disability, a reduced quality of life, and a substantial economic burden for individuals and society.

As we know, ocular hypertension is the most important risk factor for glaucoma, but its etiology is still unclear. Many molecular epidemiological studies have suggested that glaucoma is a complex multifactorial disease. Various risk factors such as diabetes, hypertension, lifestyle habits (e.g., smoking tobacco and drinking alcohol), age, and genetics play interacting roles in the development of glaucoma. Recently, certain genetic factors, including matrix metalloproteinases (MMPs), were found to be associated with glaucoma. MMPs are a group of zinc and calcium-dependent endopeptidases that are involved in extracellular matrix (ECM) homeostasis and remodeling [4, 5]. In glaucoma, pathological changes occur in the trabecular meshwork and the juxtacanalicular tissue of the chamber angle. Aqueous humor (AH) drainage is influenced by the ECM, which modulates AH outflow from the anterior chamber via the irido-corneal drainage angle to regulate IOP [6]. A recent study in animal models reported that the abnormal expression of MMPs in the AH of patients with glaucoma may influence the regulation of IOP [7]. These findings indicated that the aberrant expression of MMPs may be associated with both the development and prognosis of glaucoma.

Previous molecular research has demonstrated that genetic mutations, including single nucleotide polymorphisms (SNPs), can alter the level of gene expression or the function of gene products, thereby affecting the susceptibility of individuals to specific diseases [8, 9]. In 2006, Wang et al. [10] reported an association between SNPs in the MMP-9 gene and the risk of developing glaucoma, and suggested that the rs17576 G > A mutation maybe a risk factor in Taiwanese patients. Subsequently, considerable efforts have been made to elucidate the relationship between MMP gene polymorphisms and glaucoma risk worldwide, but conflicting results have been observed. Therefore, we conducted a comprehensive meta-analysis to evaluate the association between MMP gene polymorphisms and glaucoma risk.

## Methods

This meta-analysis was conducted according to the guidelines of Preferred Reporting Items for Systematic reviews and Meta-Analyses (PRISMA) [11, 12]. No ethical issues were involved in this study given that our data were based on published studies.

### Search strategy

Four online databases (PubMed, Embase, CNKI, Wanfang) were used to search for case control studies evaluating the association between MMPs polymorphisms and glaucoma risk published up to February 1, 2016, with the following search terms: “glaucoma,” “MMP,” “matrix metalloproteinases,” “polymorphism,” and “variant”. Manual searches of references from original studies and review articles on this topic were conducted to identify other relevant studies.

### Inclusion and exclusion criteria

The inclusion criteria for studies in our meta-analysis were as follows: (1) designed as a case control study, (2) reported an association between MMP polymorphism(s) and glaucoma risk,(3) sufficient genotype frequency to estimate odds ratios (ORs) and 95% confidence intervals (CIs), and (4) no deviation from Hardy-Weinberg equilibrium (HWE) in the genotype distribution of the control group. For results that were reported in multiple publications, only the largest or latest dataset was included. The exclusion criteria included: (1) review articles, (2) case reports, (3) results without sufficient genotype frequency data, and (4) animal model research.

### Data extraction

Two reviewers (MYW and YW) independently reviewed the full articles and collected the following characteristics: the first author’s name, publication year, study country/region, ethnicity of participants (such as Asian or Caucasian), disease subtype, genotyping method, sources of controls, and frequencies of genotypes in glaucoma cases and controls. Hardy-Weinberg equilibrium (HWE) was estimated based on the genotypes of the controls. Discrepancies were resolved by discussion between the 2 reviewers or by consulting with an expert in ophthalmology (ZY).

### Statistical analysis

Crude ORs with 95% CIs were calculated to evaluate the strength of the association between each reported MMP polymorphism and glaucoma risk. For the MMP-9 rs17576G > A polymorphism, the pooled ORs were obtained for the allele contrast (A vs. G), co-dominant (GA vs. GG,AA vs. GG), dominant (GA + AA vs. GG), and recessive (AA vs. GG + GA) models. Subgroup analyses according to disease type, ethnicity, study design, and genotyping methods were also conducted. Heterogeneity was assessed using Cochran’s Q statistic and the I^2^ method [13]. ORs estimation was calculated with a fixed-effects model (the Mantel-Haenszel method) when the P value was more than 0.10 or I^2^ was less than 50% [14]; otherwise, a random-effects model (the DerSimonian and Laird method) was adopted [15, 16]. Cumulative meta-analyses and sensitivity analyses were conducted to evaluate the stability of the results by removing each study sequentially for each polymorphism. The potential publication bias of the literature was analyzed by Egger’s linear regression and Begg’s funnel plots. Similar genetic models were also assessed for the other MMP-1, 2, and 9 variants. Statistical analysis was performed using STATA version 11.0 (Stata Corporation, College Station, TX, USA) with 2-sided *P*-values and *P* < 0.05 was considered statistically significant.

## Results

### Study characteristics

**Fig. 1:**
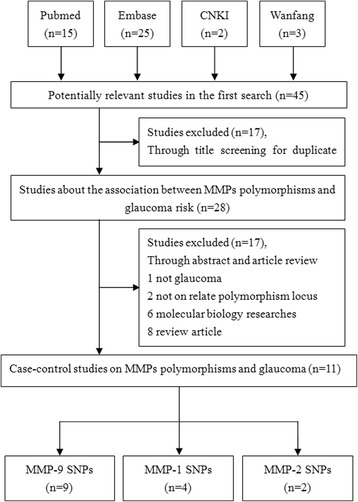
Flow diagram of the study selection process

In total, 45 related articles were identified. Seventeen of these studies were excluded through title and duplicate screening. Subsequently, 1 study was excluded for not involving glaucoma research; 2 studies without related polymorphisms locus were excluded; 6 studies were excluded for fundamental molecular biology researches; and 8 studies were excluded because they were reviews. Finally, 11 articles involving 2,388 cases and 2,319 controls met the inclusion criteria [10, 17–26] (Fig. 1). Among these included articles, 9 studies focused on associations between MMP-9 polymorphisms (rs17576 G > A, rs17577 G > A, rs3918249 C > T, rs3918242 C > T, rs3918254 C > T, rs3787268 G > A) and glaucoma [10, 17–24] (Table 1), 4 articles on associations between an MMP-1 polymorphism (rs1799750 1G > 2G) and glaucoma [19, 22, 24, 25] (Table 2), and 2 articles on associations between an MMP-2 polymorphism (rs243865C > T) and glaucoma [19, 26] (Table 2).

**Table 1:** Characteristics of case–control studies on MMP-9 polymorphisms and glaucoma risk

First author	Year	Country	Racial/descent	Type	Genotyping	Case	Control	Genotype distribution						P for HWE	Source of controls
								Case			Control				
rs17576 G > A								G/G	G/A	A/A	G/G	G/A	A/A		
Wang	2006	China	Asian	PACG	Applied Biosystems	78	86	17	17	44	38	33	15	0.11	Hospital-based
Aung	2008	Singaporean	Asian	PACG	Applied Biosystems	217	83	135	69	13	48	31	4	0.72	Healthy-based
Cong	2009	China	Asian	PACG	PCR-RFLP	211	205	119	76	16	124	75	6	0.18	Healthy-based
Mossböck1	2010	Austria	Caucasian	POAG	PCR-RFLP	322	248	42	141	139	26	120	102	0.28	Hospital-based
Mossböck2	2010	Austria	Caucasian	XGF	PCR-RFLP	202	248	31	83	88	26	120	102	0.28	Hospital-based
Awadalla	2011	Australian	Caucasian	PACG	Sequenom	104	268	18	49	37	27	109	132	0.52	Population-based
Shi	2013	China	Asian	PACG	Applied Biosystems	231	306	133	78	20	164	113	29	0.15	Population-based
Micheal1	2013	Pakistan	Asian	PACG	PCR-RFLP	82	118	35	32	15	25	53	40	0.34	Healthy-based
Micheal2	2013	Pakistan	Asian	POAG	PCR-RFLP	112	118	38	48	26	25	53	40	0.34	Healthy-based
rs17577 G > A								G/G	G/A	A/A	G/G	G/A	A/A		
Wang	2006	China	Asian	PACG	Applied Biosystems	78	86	62	14	2	69	15	2	0.30	Hospital-based
Awadalla	2011	Australian	Caucasian	PACG	Sequenom	106	268	73	29	4	213	51	4	0.64	Population-based
Gao	2014	China	Asian	PACG	PCR-RFLP	214	224	166	44	4	178	43	3	0.83	Hospital-based
rs3918249 C > T								C/C	C/T	T/T	C/C	C/T	T/T		
Awadalla	2011	Australian	Caucasian	PACG	Sequenom	106	267	19	49	38	27	109	131	0.54	Population-based
Shi	2013	China	Asian	PACG	Applied Biosystems	231	306	133	79	19	160	116	30	0.19	Population-based
Gao	2014	China	Asian	PACG	PCR-RFLP	213	221	91	109	13	104	103	14	0.08	Hospital-based
rs3918242 C > T								C/C	C/T	T/T	C/C	C/T	T/T		
Micheal1	2013	Pakistan	Asian	PACG	PCR-RFLP	82	118	56	25	1	74	37	7	0.42	Healthy-based
Micheal2	2013	Pakistan	Asian	POAG	PCR-RFLP	112	118	70	40	2	74	37	7	0.42	Healthy-based
Markiewicz	2013	Poland	Caucasian	POAG	PCR-RFLP	255	256	166	83	6	195	56	5	0.68	Hospital-based
rs3918254 C > T								C/C	C/T	T/T	C/C	C/T	T/T		
Awadalla	2011	Australian	Caucasian	PACG	Sequenom	106	283	105	1	0	282	1	0	0.98	Population-based
Gao	2014	China	Asian	PACG	PCR-RFLP	214	220	125	85	4	122	81	17	0.49	Hospital-based
rs3787268 G > A								G/G	G/A	A/A	G/G	G/A	A/A		
Awadalla	2011	Australian	Caucasian	PACG	Sequenom	106	267	64	35	7	174	84	9	0.77	Population-based
Gao	2014	China	Asian	PACG	PCR-RFLP	207	220	82	112	13	89	108	23	0.24	Hospital-based

HWE in control

Test for heterogeneity

*NA* Not available

*AB* Applied Biosystems

*PCR-RFLP* Polymerase chain reaction-restriction fragment length polymorphism

*PACG* Primary angle-closure glaucoma

*POAG* Primary open angle glaucoma

*XGF* Exfoliation glaucoma

**Table 2:** Characteristics of included studies on MMP-1 rs1799750 1G > 2G and MMP-2 rs243865C > T polymorphisms and glaucoma risk

First author	Year	Country	Racial/descent	Type	Genotyping	Case	Control	Genotype distribution						P for HWE	Source of controls
								Case			Control				
rs1799750 1G > 2G								1G/1G	1G/2G	2G/2G	1G/1G	1G/2G	2G/2G		
Tsironi	2009	Greece	Caucasian	XFG	PCR-RFLP	92	214	39	42	11	65	110	39	0.53	Hospital-based
Mossböck1	2010	Austria	Caucasian	POAG	PCR-RFLP	322	248	42	141	139	26	120	102	0.28	Hospital-based
Mossböck2	2010	Austria	Caucasian	XFG	PCR-RFLP	202	248	31	83	88	26	120	102	0.28	Hospital-based
Markiewicz	2013	Poland	Caucasian	POAG	PCR-RFLP	255	256	93	77	85	94	113	49	0.15	Hospital-based
Micheal1	2013	Pakistan	Asian	PACG	PCR-RFLP	82	118	25	36	21	53	45	20	0.06	Healthy-based
Micheal2	2013	Pakistan	Asian	POAG	PCR-RFLP	112	118	27	49	36	53	45	20	0.06	Healthy-based
rs243865C > T								C/C	C/T	T/T	C/C	C/T	T/T		
Mossböck1	2010	Austria	Caucasian	POAG	PCR-RFLP	322	248	187	111	24	138	88	22	0.15	Hospital-based
Mossböck2	2010	Austria	Caucasian	XFG	PCR-RFLP	202	248	107	80	15	138	88	22	0.15	Hospital-based
Kaminska	2014	Poland	Caucasian	POAG	PCR-RFLP	268	311	159	89	20	175	123	13	0.13	Hospital-based

HWE in control

Test for heterogeneity

*PCR-RFLP* Polymerase chain reaction-restriction fragment length polymorphism

*PACG* Primary angle-closure glaucoma

Five studies involved Asian populations [10, 17, 18, 21, 23] and 6 studies involved Caucasian populations [19, 20, 22, 24–26]. No study deviated from HWE. The detailed characteristics of the selected studies are summarized in Tables 1 and 2.

### Meta-analysis

#### Association between MMP-9 polymorphisms with glaucoma risk

**Fig. 2:**
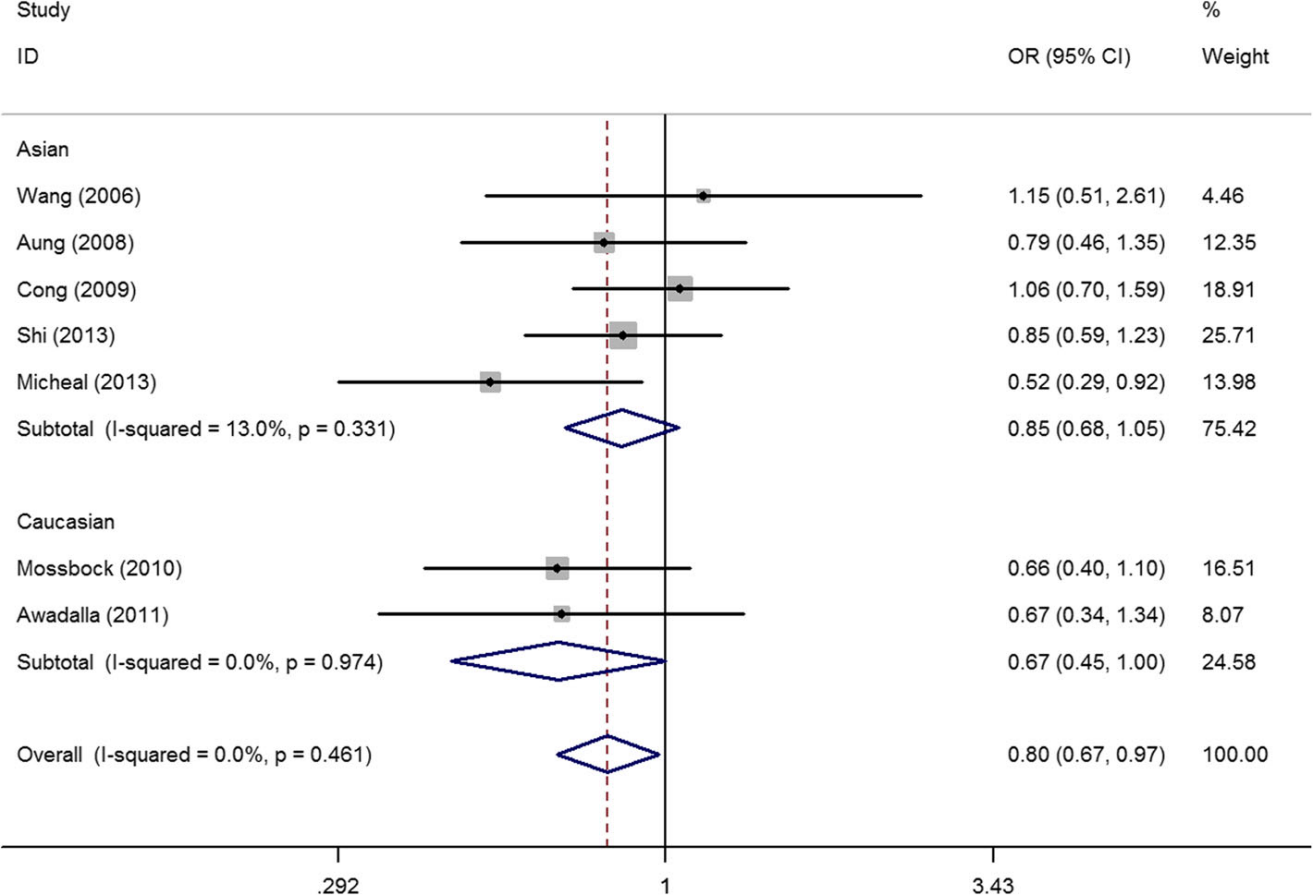
Calculated OR and 95% CIs for the associations between MMP-9 rs17576 G > A polymorphism and glaucoma risk in the GA vs. GG model

With regards to MMP-9 gene polymorphisms and glaucoma risk, 9 articles (reporting a total of 11 case–control studies) involving 2,028 cases and 1,794 controls were included in our analyses of the 6 most commonly reported SNP loci. Overall, significant associations were only found between MMP-9 rs17576 G > A polymorphism (GA vs. GG: OR = 0.80, 95%CI = 0.67-0.97, *P* = 0.02, I^2^ = 0%), MMP-9 rs3918249 C > T polymorphism (TT vs. CC + CT: OR = 0.71, 95%CI = 0.51-0.98, *P* = 0.04, I^2^ = 0%) and glaucoma risk. Furthermore, in the subsequent analyses based on disease type, ethnicity, control design and genotyping methods, a significant protective effect against glaucoma risk was observed for MMP-9 rs17576 G > A in Caucasian populations (GA vs. GG: OR = 0.67, 95%CI = 0.45-1.00, *P* = 0.05, I^2^ = 0 (Fig. 2); GA + AA vs. GG: OR = 0.66, 95%CI = 0.45-0.97, *P* = 0.03, I^2^ = 0%) (Table 3). No significant association was found between other MMP-9 polymorphisms and glaucoma risk.

**Table 3:** Summary ORs and 95% CI of MMP-9 polymorphisms and glaucoma risk

	OR	95% CI	*P*	*I*2 (%)^a^	OR	95% CI	*P*	*I*2 (%)^a^	OR	95% CI	*P*	*I*2 (%)^a^	OR	95% CI	*P*	*I*2 (%)^a^	OR	95% CI	*P*	*I*2 (%)^a^
rs17576 G > A		A vs. G				GA vs. GG				AA vs. GG				GA + AA vs. GG				AA vs. GG + GA		
Total	1.10	0.80-1.51	0.56	85.1	0.80	0.67-0.97	0.02	0	1.07	1.07 0.52-2.19	0.86	85.5	0.87	0.60-1.24	0.44	73.7	1.23	0.69-2.18	0.48	85.5
Type																				
PACG	1.01	0.62-1.62	0.98	90.6	0.83	0.68-1.03	0.09	16.3	1.10	0.43-2.86	0.84	88.0	0.88	0.56-1.37	0.57	78.9	1.25	0.56-2.85	0.58	87.7
POAG	0.82	0.51-1.26	0.34	75.8	0.67	0.44-1.01	0.06	0	0.62	0.23-1.61	0.16	54.7	0.66	0.44-0.97	0.03	0	0.84	0.46-1.52	0.56	68.7
XFG	0.95	0.72-1.25	0.71	NA	0.58	0.32-1.05	0.07	NA	0.90	0.50-1.62	0.29	NA	0.65	0.37-1.13	0.13	NA	1.10	0.76-1.61	0.60	NA
Ethnicity																				
Asian	1.27	0.83-1.96	0.27	86.4	0.85	0.68-1.50	0.13	13.0	1.41	0.49-4.06	0.52	88.6	0.98	0.62-1.56	0.94	79.5	1.55	0.60-4.01	0.34	88.2
Caucasian	0.80	0.50-1.22	0.30	77.5	0.67	0.45-1.00	0.05	0	0.61	0.33-1.12	0.11	52.0	0.66	0.45-0.97	0.03	0	0.81	0.43-1.53	0.52	81.0
Design																				
Heal-B	1.08	0.88-1.33	0.46	0	0.79	0.52-1.18	0.25	50.1	1.00	0.26-3.81	1.00	84.7	0.78	0.44-1.37	0.38	76.5	1.14	0.39-3.37	0.81	79.0
Hosp-B	1.83	0.51-6.54	0.35	96.0	0.77	0.50-1.17	0.22	20.7	2.22	0.28-17.62	0.45	94.6	1.40	0.37-5.35	0.62	90.3	2.51	0.46-13.57	0.27	94.7
Pop-B	0.76	0.55-1.05	0.10	57.2	0.81	0.58-1.12	0.20	0	0.61	0.31-1.22	0.16	54.7	0.73	0.48-1.12	0.15	35.3	0.68	0.47-0.98	0.04	30.6
Genotyping																				
AB	1.40	0.62-3.20	0.42	92.9	0.86	0.65-1.15	0.32	0	1.87	0.48-7.29	0.37	87.3	1.20	0.62-2.30	0.59	80.5	1.93	0.52-7.18	0.32	88.0
PCR-RFLP	1.05	0.89-1.23	0.57	0	0.74	0.48-1.13	0.16	55.7	0.86	0.32-2.29	0.76	84.1	0.74	0.42-1.30	0.30	77.1	1.05	0.51-2.16	0.89	81.2
rs17577 G > A		A vs. G				GA vs. GG				AA vs. GG				GA + AA vs. GG				AA vs. GG + GA		
Total	1.30	0.98-1.72	0.07	12.0	1.26	0.91-1.74	0.16	0	1.79	0.71-4.48	0.22	0	1.30	0.95-1.78	0.10	2.3	1.70	0.68-4.24	0.26	0
Ethnicity																				
Asian	1.11	0.78-1.58	0.58	0	1.08	0.72-1.62	0.70	0	1.31	0.39-4.34	0.66	0	1.10	0.78-1.58	0.64	0	1.29	0.39-4.27	0.68	0
Design																				
Hosp-B	1.11	0.78-1.58	0.58	0	1.08	0.72-1.62	0.70	0	1.31	0.39-4.34	0.66	0	1.10	0.78-1.58	0.64	0	1.29	0.39-4.27	0.68	0
rs3918249 C > T		T vs. C				CT vs. CC				TT vs. CC				CT + TT vs. CC				TT vs. CC + CT		
Total	0.84	0.62-1.14	0.27	67.9	0.91	0.65-1.28	0.59	41.0	0.68	0.46-1.02	0.06	39.9	0.84	0.56-1.47	0.40	63.1	0.71	0.51-0.98	0.04	0
Ethnicity																				
Asian	0.96	0.73-1.25	0.75	43.9	0.99	0.68-1.45	0.96	50.9	0.86	0.53-1.40	0.55	0	0.97	0.66-1.42	0.89	55.0	0.87	0.54-1.40	0.58	0
Design																				
Heal-B	0.74	0.56-0.98	0.03	41.6	0.77	0.56-1.07	0.12	0	0.57	0.31-1.04	0.07	40.9	0.73	0.54-0.99	0.05	32.7	0.66	0.46-0.95	0.03	0
rs3918242 C > T		T vs. C				CT vs. CC				TT vs. CC				CT + TT vs. CC				TT vs. CC + CT		
Total	1.13	0.60-2.13	0.70	82.5	1.37	0.82-2.28	0.23	61.7	0.62	0.11-3.33	0.58	70.5	1.27	0.68-2.36	0.46	78.5	0.57	0.12-2.68	0.48	65.4
Type																				
POAG	1.20	0.69-2.06	0.52	73.0	1.51	1.09-2.08	0.01	31.8	0.72	0.16-3.23	0.67	56.2	1.36	0.81-2.28	0.24	59.7	0.65	0.16-2.64	0.55	50.1
Design																				
Heal-B	0.81	0.57-1.13	0.22	0	1.02	0.98-1.54	0.91	0	0.25	0.07-0.92	0.04	0	0.92	0.60-1.34	0.60	0	0.25	0.07-0.89	0.03	0
rs3918254 C > T		T vs. C				CT vs. CC				TT vs. CC				CT + TT vs. CC				TT vs. CC + CT		
Total	0.80	0.58-1.09	0.15	0	1.04	0.71-1.54	0.83	0	NA	NA	NA	NA	0.90	0.62-1.32	0.60	0	NA	NA	NA	NA
rs3787268 G > A		A vs. G				GA vs. GG				AA vs. GG				GA + AA vs. GG				AA vs. GG + GA		
Total	1.06	0.78-1.43	0.72	40.3	1.13	0.83-1.54	0.44	0	1.08	0.32-3.62	0.90	72.7	1.11	0.83-1.50	0.49	0	1.02	0.30-3.51	0.97	75

^a^ Test for heterogeneity

*Heal-B* Healthy-based

*Hosp-B* Hospital controls

*Pop-B* Population controls

*NA* Not available

*AB* Applied Biosystems

*PCR-RFLP* Polymerase chain reaction-restriction fragment length polymorphism

*PACG* Primary angle-closure glaucoma

*POAG* Primary open angle glaucoma

**Fig. 3:**
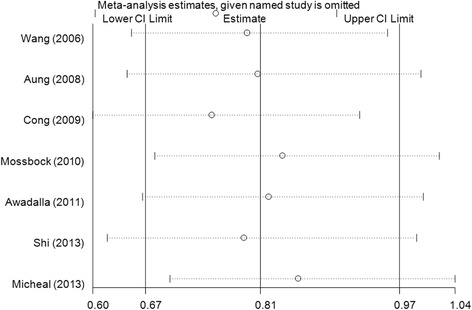
Sensitivity analysis via deletion of each individual study reflects the relative influence of each individual dataset on the pooled ORs in the GA vs. GG model of MMP-9 rs17576 G > A polymorphism and glaucoma risk

**Fig. 4:**
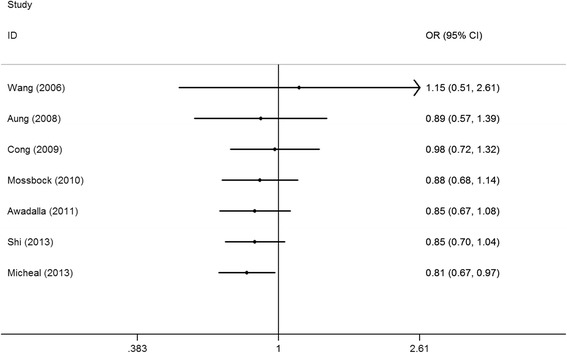
Cumulative meta-analyses according to publication year in the GA vs. GG model of MMP-9 rs17576 G > A polymorphism and glaucoma risk

**Fig. 5:**
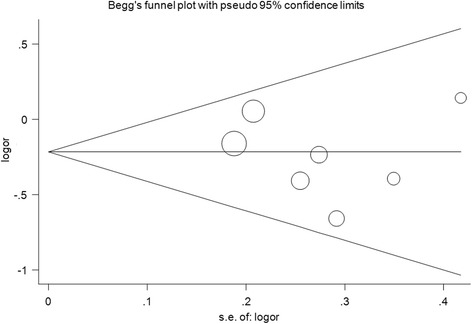
Funnel plot analysis to detect publication bias for GA vs. GG model of MMP-9 rs17576 G > A polymorphism and glaucoma risk. Circles represent the weight of the studies

Sensitivity analyses were conducted by deleting each included study step by step in MMP-9 rs17576 G > A polymorphism. No single study qualitatively changed the pooled ORs when removed, indicating that the results of this meta-analysis are stable (Fig. 3 for GA vs. GG model). Cumulative analysis showed that the protective effect increased gradually with the increase of sample size by publication date in MMP-9 rs17576 G > A polymorphism (Fig. 4 for GA vs. GG model). Funnel plots were performed to assess the potential for publication bias, and no evidence of asymmetry was found (Fig. 5 for GA vs. GG model). This result was further supported by analysis using Egger’s test (A vs. G: *P* = 0.30; GA vs. GG: *P* = 0.57; AA vs. GG: *P* = 0.28; GA + AA vs. GG: *P* = 0.95; AA vs. GG + GA, *P* = 0.43), indicating that there was no detectable publication bias.

#### Association between MMP-1 and MMP-2 polymorphisms with glaucoma risk

Four articles (reporting a total of 6 case control studies) involving 1,065 cases and 836 controls and 2 articles (reporting a total of 3 case control studies) with 792 cases and 559 controls were included in our meta-analysis of the MMP-1 rs1799750 1G > 2G and MMP-2 rs243865 C > T polymorphisms and glaucoma risk. No significant association was found for all models for these 2 SNP loci (Table 4). Subgroup analyses based on disease type and control design were also conducted, and no significant association was found.

**Table 4:** Summary ORs and 95% CI of MMP-1 rs1799750 1G > 2G and MMP-2 rs243865C > T polymorphisms and glaucoma risk

	OR	95% CI	*P*	*I* ^2^ (%)^a^	OR	95% CI	*P*	*I* ^2^ (%)^a^	OR	95% CI	*P*	*I* ^2^ (%)^a^	OR	95% CI	*P*	*I* ^2^ (%)^a^	OR	95% CI	*P*	*I* ^2^ (%)^a^
rs1799750 1G > 2G	2G vs. 1G				1G/2G vs. 1G/1G				2G/2G vs. 1G/1G				1G/2G +2G/2G vs. 1G/1G				2G/2G vs. 1G/1G +1G/2G			
Total	1.14	0.79-1.64	0.49	85.0	0.86	0.52-1.41	0.55	75.7	1.20	0.59-2.43	0.61	83.4	0.99	0.59-1.69	0.98	81.8	1.35	0.83-2.21	0.23	77.2
Type																				
PACG	1.61	1.07-2.42	0.02	NA	1.70	0.89-3.24	0.11	NA	2.33	1.03-4.83	0.04	NA	1.86	1.03-3.37	0.04	NA	1.69	0.85-3.37	0.14	NA
POAG	137	0.94-2.01	0.11	81.7	1.00	0.51-1.93	0.99	79.7	1.69	0.81-3.50	0.16	80.0	1.24	0.66-2.33	0.50	8.07	1.69	1.01-2.81	0.05	7.54
XFG	0.82	0.59-1.12	0.23	51.6	0.61	0.41-0.91	0.02	0	0.62	0.38-0.99	0.05	0	0.62	0.42-0.90	0.01	0	0.89	0.51-1.56	0.69	5.17
Design																				
Heal-B	1.87	1.34-2.61	<0.01	NA	1.93	1.14-3.26	0.02	NA	2.90	1.54-5.49	<0.01	NA	2.23	1.37-3.61	<0.01	NA	2.04	1.15-3.61	0.02	NA
Hosp-B	0.98	0.69-1.38	0.91	79.4	0.67	0.51-0.88	<0.01	0	0.91	0.44-1.90	0.82	80.4	0.81	0.63-1.04	0.10	36.1	1.18	0.65-2.16	0.58	81.7
rs243865 C > T	T vs. C				CT vs. CC				TT vs. CC				CT + TT vs. CC				TT vs. CC + CT			
Total	0.78	0.82-1.17	0.79	0	0.91	0.72-1.15	0.43	3.4	1.14	0.57-2.28	0.71	56.3	0.94	0.75-1.17	0.55	0	1.19	0.54-.2.62	0.66	67.4
Type																				
POAG	95	0.79-1.15	0.62	0	0.86	0.67-1.10	0.23	0	1.14	0.55-2.35	0.73	56.8	0.89	0.71-1.13	0.35	0	1.21	0.55-1.65	0.64	64.5

^a^ Test for heterogeneity

*Heal-B* Healthy-based

*Hosp-B* Hospital controls

*NA* Not available

*PACG* Primary angle-closure glaucoma

*POAG* Primary open angle glaucoma

*XGF* Exfoliation glaucoma

## Discussion

MMPs are classified as a large family of zinc-containing proteases and have been suggested to be important mediators in the pathogenesis of various diseases. MMPs, which are antagonized by tissue inhibitors of metalloproteinases (TIMPs), can degrade and remodel ECM molecules, thereby influencing cellular activities and maintaining the homeostasis of theepithelialbasementmembrane [27, 28]. The abnormal expression of MMPs can disturb the proteolytic balance and result in a number of pathologic conditions such as inflammatory diseases [29], oropharyngeal cancer [30], coronary heart disease [31], and respiratory abnormalities [32]. In the development of glaucoma, an imbalance between MMPs and TIMPs may impair ECM turnover in the trabecular meshwork and increase the resistance to AH outflow, which may eventually lead to raised IOP and glaucoma [33].

SNPs are the most common type of genetic mutation and have been associated with altered disease susceptibility through changes in gene transcription and expression as well as amino acid substitutions. In 2006, Wang et al. conducted the first case control study investigating the potential association between MMP-9 SNPs and primary angle-closure glaucoma. Significant differences in the frequencies of the MMP-9 rs17576 G > A SNP genotypes were found between the glaucoma and healthy control groups, which suggested an increased risk for glaucoma in the Chinese population according to a dominant model (OR = 2.84, 95%CI = 1.63-5.64, *P* = 0.03). Subsequently, more epidemiological studies were conducted, with inconsistent and even contradictory results.

In this meta-analysis, published research data were pooled and analyzed to investigate a specific research question. Pooling datasets reduces the random error that can occur with small sample sizes. This retrospective review indicated that an inadequate number of studies, small sample sizes and limited ethnic diversity, contributed to their conflicting findings. To evaluate the evidence for the potential association between MMPs and glaucoma susceptibility, we conducted this meta-analysis using 2,388 cases and 2,319 controls from 11 publications. Overall, our analysis indicated that MMP-9 polymorphisms (rs17576 G > A and rs3918249 C > T) conferred different significantly susceptibility to glaucoma. Stratified analysis by ethnicity, control design, and genotyping method were performed to estimate the association for each subgroup. The subgroup analysis according to ethnicity demonstrated a significant protective association between the MMP-9 rs17576 G > A polymorphism and glaucoma susceptibility in the Caucasian population. Thus, ethnic differences may be the most important factor underlying differences in glaucoma susceptibility between the Asian and Caucasian populations. MMP-9 rs17576 polymorphism is locate in exon 6 of MMP-9 gene with a nucleic acid substitution from G to A, and this mutation located fibronectin type II domains that presumably enhance substrate binding [34, 35]. Although no experimental research on this polymorphism was reported. The conversion from the positively charged amino acid arginine to uncharged glutamine may influence the activity of this enzyme and affect the glaucoma susceptibility [36, 37]. As we know, the pathogenesis of primary open-angle glaucoma (POAG)、primary closure-angle glaucoma (PACG) and exfoliation syndrome (XFG) are not identical. PACG is characterized by the adhesion between the peripheral iris and trabecular meshwork, resulting in the inability of the aqueous fluid to flow out of the aqueous humor. POAG is thought to be a common glaucoma, accompanied with optic neuropathy and corresponding visual field progressive damage with an open situation of anterior chamber angle. The etiology of XFG is still unknown,which generally believed to be a systemic disorder of eye condition. They are caused by intermittent or persistent elevation of IOP, resulting in damage to the eye tissue and visual function. In this meta-analysis,subgroup analysis of POAG、PACG and XPF risk were conducted in only two polymorphism loci due to the small sample size of included studies. Regrettably, this study failed to find a direct correlation between the MMPs genetic polymorphisms and the three sub-diseases.

To the best of our knowledge, this is the first comprehensive meta-analysis evaluating the potential association between MMP gene polymorphisms and glaucoma susceptibility. Some limitations of this meta-analysis were inevitable and should be addressed. First, the number of included studies and the amount of data available were limited, which constrained the statistical power. Second, heterogeneity was observed in the data for some SNP loci, which further hindered our ability to confidently identify any potential associations. Third, due to the deficiency of available data, it was not possible to study the interactions between MMP gene SNPs and glaucoma risk factors, such as haplotype, tobacco smoking, alcohol drinking, hypertension, and diabetes mellitus.

Despite these limitations, the findings further enhance our understanding of the potential associations between MMP gene polymorphisms and glaucoma susceptibility. Positive aspects of the analysis were also identified. First, the distributions of genotypes in the control subjects for all of the investigated SNPs conformed to HWE. Second, evaluation using Egger’s test and Begg’s funnel plots did not show significant publication bias. Third, a large amount of heterogeneity was alleviated through subgroup analyses.

## Conclusion

In conclusion, our meta-analysis indicated that the MMP-9 rs17576 G > A polymorphism maybe an important protective factor against glaucoma, especially in the Caucasian population. Moreover, the present findings highlight the need for further investigations of the potential associations between MMP gene polymorphisms and glaucoma, which should ideally large sample sizes and multiple ethnic groups.

## Abbreviations

95% CIs: 95% confidence intervals
AH: Aqueous humor
CIs: Confidence intervals
ECM: Extracellular matrix
HWE: Hardy-Weinberg equilibrium
IOP: Intraocular pressure
MMPs: Matrix metalloproteinases
ORs: Odds ratios
PRISMA: Preferred reporting items for systematic reviews and meta-analyses
SNPs: Single nucleotide polymorphisms
TIMPs: Tissue inhibitors of metalloproteinases
